# Rare clinical phenotype of filaminopathy presenting as restrictive cardiomyopathy and myopathy in childhood

**DOI:** 10.1186/s13023-022-02477-5

**Published:** 2022-09-14

**Authors:** A. Muravyev, T. Vershinina, P. Tesner, G. Sjoberg, Yu. Fomicheva, N. Novák Čajbiková, A. Kozyreva, S. Zhuk, E. Mamaeva, S. Tarnovskaya, J. Jornholt, P. Sokolnikova, T. Pervunina, E. Vasichkina, T. Sejersen, A. Kostareva

**Affiliations:** 1 Almazov National Medical Research Centre, St Petersburg, Russia 197341; 2 Department of Biology and Medical Genetics, 2nd Faculty of Medicine, Charles University and University Hospital Motol, Prague, Czech Republic; 3 Department of Women’s and Children’s Health, Center for Molecular Medicine, Karolinska Institute, 17176 Stockholm, Sweden

**Keywords:** Genes, Mutation, *FLNC*-associated phenotype, Restrictive cardiomyopathy, Congenital myopathy, Rare clinical phenotype, Childhood, Unfavourable prognosis

## Abstract

**Background:**

*FLNC* is one of the few genes associated with all types of cardiomyopathies, but it also underlies neuromuscular phenotype. The combination of concomitant neuromuscular and cardiac involvement is not often observed in filaminopathies and the impact of this on the disease prognosis has hitherto not been analyzed.

**Results:**

Here we provide a detailed clinical, genetic, and structural prediction analysis of distinct *FLNC*-associated phenotypes based on twelve pediatric cases. They include early-onset restrictive cardiomyopathy (RCM) in association with congenital myopathy. In all patients the initial diagnosis was established during the first year of life and in five out of twelve (41.7%) patients the first symptoms were observed at birth. RCM was present in all patients, often in combination with septal defects. No ventricular arrhythmias were noted in any of the patients presented here. Myopathy was confirmed by neurological examination, electromyography, and morphological studies. Arthrogryposes was diagnosed in six patients and remained clinically meaningful with increasing age in three of them. One patient underwent successful heart transplantation at the age of 18 years and two patients are currently included in the waiting list for heart transplantation. Two died due to congestive heart failure. One patient had ICD instally as primary prevention of SCD. In ten out of twelve patients the disease was associated with missense variants and only in two cases loss of function variants were detected. In half of the described cases, an amino acid substitution A1186V, altering the structure of IgFLNc10, was found.

**Conclusions:**

The present description of twelve cases of early-onset restrictive cardiomyopathy with congenital myopathy and FLNC mutation, underlines a distinct unique phenotype that can be suggested as a separate clinical form of filaminopathies. Amino acid substitution A1186V, which was observed in half of the cases, defines a mutational hotspot for the reported combination of myopathy and cardiomyopathy. Several independent molecular mechanisms of *FLNC* mutations linked to filamin structure and function can explain the broad spectrum of *FLNC*-associated phenotypes. Early disease presentation and unfavorable prognosis of heart failure demanding heart transplantation make awareness of this clinical form of filaminopathy of great clinical importance.

**Supplementary Information:**

The online version contains supplementary material available at 10.1186/s13023-022-02477-5.

## Introduction

The availability of high throughput sequencing and its extensive use in clinical medicine has brought several newly acknowledged genes in focus of clinical genetics. One of these genes is the *FLNC* gene encoding the filamin C protein. In the era of Sanger sequencing, it was not actively studied due to its large size, it consists of 48 exons and encodes the actin-binding protein of 291 kDa. In the first studies dated to 2005, *FLNC* was found to be associated with the development of myofibrillar myopathy (MFM), a neuromuscular disorder characterized by excessive intracellular protein aggregates accumulation. In the following years, *FLNC* pathogenic variants were also described in patients with distal myopathies (DM) [15, 25, 26, 29, 46, 56]. On the other hand, cardiovascular phenotype was rarely reported in association with *FLNC*-related myopathies, and the first description of a series of *FLNC*-related isolated cardiomyopathies was published almost ten years after the first studies, in 2014 [53]. Subsequently, the application of next-generation sequencing technology resulted in multiple reports on the role of *FLNC* in different types of cardiomyopathies [7, 8, 33, 53]. Currently, isolated cardiomyopathy phenotype is well recognized as one of the main clinical entities of filaminopathies [16].

At present, variants in *FLNC* are associated with either neuromuscular phenotype (MFM or DM) or different types of cardiomyopathies; dilated, arrhythmogenic, hypertrophic, or restrictive [1, 18]. Importantly, each of the mentioned clinical conditions is associated with distinct molecular pathomechanisms. Loss of function (LOF) *FLNC* variants lead to protein haploinsufficiency often through nonsense-mediated RNA decay and cause dilated and arrhythmogenic cardiomyopathies, or, less often, DM [7, 19]. In contrast, *FLNC* variants, leading to protein instability and formation of intracellular aggregates, are associated with MFM, hypertrophic or restrictive cardiomyopathy [8, 43, 44].

Thus, the broad spectrum of *FLNC*-associated phenotypes can be explained by the existence of independent molecular mechanisms of mutations action that affect protein structure and functions (for review, see Mao et al. [30]). The knowledge of these fine molecular mechanisms, their genetic bases, and clinical consequences could potentially result in personalized treatment options, which nowadays are being developed for many inherited diseases, including neuromuscular disorders and cardiomyopathies [5, 12, 40].

Currently, *FLNC,* together with *TTN,* are the only two genes associated with all types of cardiomyopathies, including arrhythmogenic (ACM), dilated (DCM), hypertrophic (HCM), and restrictive (RCM) ones. Such association with completely different myocardial phenotypes highlights the variety of filamin C intracellular functions determined by the complex structure of the protein and the presence of diverse functional domains affected by mutations.

According to Ader et al., *FLNC*-associated cardiomyopathies account for only a small proportion of all cardiomyopathy genetic variants, namely 1.3% of HCM, 3% of DCM, and 8% of RCM [1]. Although these numbers depend on the studied population, the present and similar reports generally demonstrate the overall low prevalence of *FLNC* mutations among patients with cardiomyopathies [6, 33]. However, even with such low prevalence, increasing knowledge on *FLNC*-associated cardiomyopathies has contributed to conclusions regarding the genotype–phenotype correlation. Thus, *FLNC* LOF variants are now commonly accepted as determinants of more severe prognosis and high risk of sudden cardiac death in DCM and ACM [2, 33, 42]. In contrast, much less is known regarding *FLNC*-linked HCM and RCM. The scarcity of data can possibly be explained by the low frequency of HCM phenotype caused by *FLNC* variants and the low frequency of RCM in itself. However, these cases, especially with severe diastolic dysfunction and restrictive phenotype, are associated with high risk of developing heart failure and constitute indication for heart transplantation.

Very limited information is available regarding *FLNC*-associated phenotypes presenting in childhood [37, 44, 61]. Based on the available published reports, the combination of concomitant neuromuscular and cardiac involvement is not often observed in filaminopathies. The impact of this combination on the disease prognosis in children and adults has not been analyzed yet.

We recently described four cases of distinct *FLNC*-linked phenotype, including early-onset RCM in association with congenital myopathy [24]. Subsequently, we have succeeded to analyse more such cases (totalling 12 including four already published) reported from several clinical centres from different countries. The present study provides a more detailed clinical, genetic, and structural prediction analysis of this form of filaminopathy based on an extended number of patients. We believe that our results demonstrate the existence of the distinct clinical entity of filaminopathy and underscore the role of the *FLNC* gene in the development of pediatric RCM combined with myopathy.

## Material and methods

### Patient cohort

The study represents a meta-analysis of previously reported and newly diagnosed pediatric cases of *FLNC*-associated RCM and/or myopathy. Patients 1–4 were previously reported by Kiselev et al. [24]. Patients were recruited in Almazov National Medical Research Centre (St. Petersburg, Russia), Karolinska Institute (Stockholm, Sweden), and University Hospital Motol (Prague, Czech Republic). The study was performed according to the Declaration of Helsinki, and approval was obtained from Almazov National Medical Research Centre Ethical Committee, Karolinska Institute Ethical Review Board, and Ethical committee of University Hospital Motol. Written informed consent was obtained from all subject representatives prior to the investigation.

The diagnosis of RCM was based on the WHO/International Society and Federation of Cardiology Task Force clinical criteria and classified according to the European Society of Cardiology classification of cardiomyopathies [36]. In pediatric patients, the diagnosis was based on the following echocardiography features: atrial dilation in combination with normal or nearly normal left ventricular size and preserved or nearly preserved systolic function (left ventricular end-diastolic dimension z-score ≤ 3, fractional shortening ≥ 0.25). Severe diastolic dysfunction was established based on transmitral spectral Doppler restrictive filling pattern (accentuated early diastolic velocity with low or absent late filling velocity with E/A ratio > 1.5, E wave deceleration time < 150 ms, and short isovolumic relaxation time < 60 ms). Echocardiographic parameters of the right and left atria were calculated in the apical four-chamber view; cardiac MRI was performed in four patients.

In order to investigate presence of neuromuscular symptoms, each patient underwent a full neurological examination, eleven out of twelve patients underwent EMG. Morphological examination was performed on striated muscle tissue samples from two patients.

### Genetic testing

Target and whole-exome sequencing were performed as described previously [22]. For Patients 1–5 and 8, whole-exome sequencing was performed using a SureSelect Human All Exon V6 r2 (60 Mbp) target enrichment kit (Agilent Technologies, Santa Clara, CA, USA) with an Illumina HiSeq instrument and SBSv4 chemistry (Illumina, San Diego, CA, USA). For Patients 6, 7, 9, 10, 11, 12, a targeted panel of 172 cardiomyopathy-associated genes was studied using the SureSelect Target Enrichment System (Agilent; Waldbronn, Germany) with an Illumina MiSeq instrument (see Additional file 1). Alignment, data processing, and variant calling were performed according to GATK BestPractice recommendations using hg19 human genome reference. Variant annotation was performed using ANNOVAR (Philadelphia, PA, USA). The variant filtering and data processing were performed as described previously [22]. Briefly, variants were filtered based on their functional consequences and further, nonsynonymous single-nucleotide variants, insertions, deletions, and stop-gain mutations were processed based on their pathogenicity predicted by various prediction programs, such as CADD and SIFT. Finally, bidirectional Sanger sequencing was performed to validate all reported variants.

### Structure analysis of filamin C Ig domains

Domain coordinates and the FASTA sequence for the human filamin C protein were obtained from the UniProt entry Q14315. Multiple sequence alignment for immunoglobulin-like (Ig)-like domains of filamin C was calculated by Clustal Omega [47] and visualized in Jalview 2.8.2 [59]. For each column in the alignment, Jalview calculates a conservation score between 0 (no conservation) and 10 (high conservation). Structures of FLNC Ig-like domains were built with the SWISS-MODEL server [58] using known human filamin A crystal structures 4m9p [45] and 2j3s [28] as templates. Pathogenic variants were mapped onto these structural models. Structure-based prediction method SDM [34] was employed to assess the possible impact of amino acid substitutions on the stability of Ig domains based on structural evidence. This method measures the free energy difference (DDG) upon mutation and the relative solvent-accessible area (RSA) of the mutated residue. DDG values greater than and less than zero indicate increased protein stability and destabilizing mutations, respectively. Mutated residues with RSA < 20 are considered buried, while those with RSA > 80 are highly solvent-exposed.

In silico visualization was conducted using the PyMol 2.3 software. The domain-domain interface was analyzed using a PyMol script *InterfaceResidue.py*.

### Statistics

Statistical analysis was performed using Microsoft Excel and GraphPad Prism software to illustrate data in Supporting Materials. To determine differences between groups, t-test was used. P value < 0.01 was considered significant. Plots represent means with standard deviations.

## Results

The study group included twelve children presenting with early-onset cardiomyopathy and myopathy due to *FLNC* pathogenic and likely-pathogenic variants. In all patients, the initial symptoms appeared during the first year of life and in five out of twelve (41.7%) patients, the first symptoms were observed at birth (mainly due to arthrogryposis, Table 1). The most common red flags that warranted further cardiac investigation were slow weight gain, echocardiography abnormalities associated with congenital heart disease, or heart murmurs. In one family, the familial history of cardiac or neuromuscular disorders was reported.

**Table 1:** Clinical characteristics of patients with *FLNC*-associated RCM and congenital myopathy

	Pt1	Pt2	Pt3	Pt4	Pt5	Pt6	Pt7	Pt8	Pt9	Pt10	Pt11	Pt 12
Gender	f	m	f	m	m	f	m	m	m	m	m	m
Genotype	chr7: 128485066-7:GC > CT NM_001458: **A1183L** rs1131692185	chr7: 128485076:C > T NM_001458: **A1186V** rs1114167361	chr7: 128485076:C > T NM_001458: **A1186V** rs1114167361	chr7: 128485076: C > T NM_001458: **A1186V** rs1114167361	chr7: 128485076:C > T NM_001458: **A1186V** rs1114167361	chr7: 128489034: 4926_4927del NM_001458: **splice cite loss** **−**	chr7: 128494628: G > A NM_001458: **V2297M** rs1420394583	chr7: 128485076: C > T NM_001458: **A1186V** rs1114167361	chr7: 128485076: C > T NM_001458: **A1186V** rs1114167361	chr7: 128497173: 75 bp del NM_001458: **V2522_S2546del** **−**	chr7: 128493858: G > A NM_001458: **G2151S** **−**	chr7: 128494598: A > C NM_001458: **T2287P**
ACMG classification	P (PS3, PM1, PM2, PP3, PP4)	P (PS2, PS3, PM1, PM2, PP3, PP4)	P (PS3, PM1, PM2, PP3, PP4)	P (PS2, PS3, PM1, PM2, PP3, PP4)	P (PS2, PS3, PM1, PM2, PP3, PP4)	P (PVS1, PS2, PM2, PP3)	LP (PM1, PM2, PP2, PP3, PP5)	P (PS2, PS3, PM1, PM2, PP3, PP4)	P (PS2, PS3, PM1, PM2, PP3, PP4)	P (PVS1, PS2, PM2, PP3)	LP (PM1, PM2, PP3, PP4)	LP (PM1, PM2, PP3, PP4)
Mutation status	**−**	De novo	**−**	**−**	De novo	**−**	Maternal	De novo	De novo	De novo	**−**	**−**
Initial symptoms initiated clinical contact	Slow weight gain, dyspnea	Weakness	Slow weight gain	Slow weight gain, ECG signs of enlarged atria	Arthrogryphosis at birth. Inappropriate sinus tachycardia at 6 min walk at age 15 led to first cardiac visit	Echo in 6 month—ASD	Echo in neonatal period—ASD	Stiff neck (Klippel-Feil-like), hypertonus from birth	Arthrogryposis (fixed pronation position, unable to supinate)	Echo in 6 month—VSD	Echo in neonatal period—muscle VSD Echo in 2 y.o.—atrial dilatation	4 month—Slow Weight Gain, Dyspnea. Echo in 6 month—multiple VSDs, CoA
Age of NM presentation	At birth	At birth	During first year	During first year	At birth joint contractures	During first year	During first year	At birth	At birth	During first year	During first year	4 month
Family history	**−**	**−**	**−**	**−**	**−**	**−**	+ (mother-RCM, AF)	**−** (insignificant ventricular extrasystoles in the mother)	+ (mother, RF ablation for arrhythmia)	**−**	**−**	**−**
Cardiomyopathy phenotype	RCM/HCM ASD	RCM ASD	RCM ASD	RCM VSD	RCM	RCM ASD	RCM	RCM	RCM	RCM VSD	RCM, muscle VSD	RCM, multiple VSDs, Aortic Arch hypoplasia
Age of cardiomyopathy diagnosis	6 months	1.2 y.o	3 years	1 y.o	15 y o	3 y.o	3 y.o	3.5 y.o	2 y.o	6 months	at birth	6 months
Conduction disturb	RBBB	RBBB	RBBB	**−**	AV block I	RBBB	RBBB	AV block I, RBBB	**−**	RBBB	RBBB	RBBB, AV block 1
SVT/AF	+ (1 paroxysm SVT)	**−**	**−**	+ (1 paroxysm SVT)	**−**	**−**	+ (AF)	**−**	**−**	**−**	**−**	**−**
VT	**−**	**−**	**−**	**−**	**−**	**−**	**−**	**−**	**−**	**−**	**−**	**−**
SCD	**−**	**−**	**−**	**−**	**−**	**−**	**−**	**−**	**−**	**−**	**−**	**−**
Htx list/performed	+/**−**	**−**	**−**	**−**	+/+	**−**	+/**−**	−	**−**	**−**	**−**	**−**
Cardiac Enzymes	↑CK-MB × 2 ↑Troponin × 1.5	↑CK-MB × 2–4 Normal Troponin	↑CK-MB × 2 Normal Troponin	↑CK-MB × 2.5 Normal Troponin	↑CK-MB × 1.5 Troponin n/a	Normal CK-MB Normal Troponin	↑CK-MB × 1.5 Normal Troponin	↑ CK-MB × 2.1 Troponin n/a	Normal CK-MB Troponin n/a	↑CK-MB × 1.16 Normal Troponin	Normal CK-MB Normal Troponin	Normal CK-MB Normal Troponin
NT-proBNP	↑ × 83 (10,434 pg/mL)	↑ × 25 (3157 pg/mL)	↑ × 50 (6279 pg/mL)	↑ × 21 (3116 pg/mL)	n/a	↑ × 7 (1019 pg/mL)	↑ × 17 (2174 pg/mL)	n/a	n/a	↑ × 7 (1030 pg/mL)	↑ × 40 (4986 pg/mL)	↑ × 19 (3724 pg/mL)
Cardiac MRI	Not performed	Not performed	The pericardium is not changed. Severe dilatation of both atria; ventricles are not dilated. LV ejection fraction at the lower limit of the norm. No clear pathological contrast delay	The pericardium is not changed. Severe dilatation of both atria; ventricles are not dilated. LV ejection fraction is normal. On delayed post-contrast images in the mid-apical part of the antero-septal region, minimally pronounced intramural fibrous changes are determined	Not performed	Not performed	The pericardium is not changed. The right atrium is dilated, the left atrium is moderately dilated, the ventricles are not dilated. No clear pathological contrast delay agent in the LV myocardium	Not performed	Not performed	The MRI picture may correspond to a non-compact LV myocardium. Dilation of the LA. On delayed post-contrast images in the basal parts of the antero-septal region, minimally pronounced intramural fibrous changes are determined	Not performed	Not performed
Limb-girdle weakness	+	+	+	−	+	−	+	+	−	−	−	+
Distal muscle weakness	−	+	−	−	−	+	−	−	−	−	−	+
Difficulty toe walking	+	−	−	−	−	+	−	+ (with difficulties)	−	−	−	−
Winged scapulas	−	+	+	−	−	+	+	+	+	+	−	+
Facial weakness	−	+	+	−	−	−	+	+	−	−	+	−
Ptosis	−	+	−	−	−	−	+	−	−	−	−	−
Camptodactyly	−	−	−	−	−	+ (5th fingers)	+	−	−	−	−	−
Inability to Rise from squatting Position	+	+	+	−		−	−	− (balance problems)	−	−	−	−
Arthrogryposis	+ (at birth)	+ (at birth)	−	−	+ (at birth)	−	−	+	+	−	−	+
Scoliosis	−	+	+ (severe progressive)	−	+ (severe, progressive—> scoliosis surgery)	−	+	+	−	−	+	−
Pectus deformation	−	Pectus excavatum	−	−	−	−	−	Pectus carinatum	−	−	−	−
Hip dysplasia	+	+	+	−	−	+	−	+	−	−	−	−
Torticollis	−	−	−	−	−	+	+	+	−	−	−	−
Joint contracture	−	+	−	+	+ Marked contractures shoulders. Mild contractures hips, ankles, index toes, elbows, wrists, right thumb	+ (Ankle stifness)	−	+	+ (forearm)	+ (Ankle stifness)	−	−
Hernia	White line hernia	Umbilical hernia	White line hernia	−	−	−	−	−	−	−	+	Umbilical hernia
Other	Microsomia	−	Microsomia	Growth retardation. Pes equinus Long QT	Muscle biopsy: centrally located nuclei. Cytoplasmic body myopathy	Short stature of mixednesis; Hypoglycemia, long QT	Periorbital cyanosis	Sprengler deformity, Noonan-like phenotype	Micromandibula, short neck, hyperkyphosis, pedes plani, undescended testes	Presyncope, Long QT	−	Microsomia, Pulmonary hypertension, coagulopathy, Diffuse non-toxic goiter, subclinical hypothyroidism
CNS	−	Pyramid insufficiency Clonical seizures	−	Pyramid insufficiency	−	−		−	−	−	−	−
ENMG	Diffuse myopathic pattern	Diffuse myopathic pattern Neuropathy	Diffuse myopathic pattern	Sensory polyneuropathy in the legs and arms	Not performed	Diffuse sensory neuropathy	−	Normal	−	Normal	−	Diffuse myopathic pattern
Muscle Enzymes	Normal CK ↑LDH × 1.5	↑CK × 1.5–4 ↑LDH × 1.5	↑CK × 1.2 ↑LDH × 1.5	↑CK × 1.32 ↑LDH × 1.84	Transaminases normal	Normal CK ↑LDH × 1.45	Normal CK Normal LDH	↑CK × 2.8, ↑LDH × 1.4, ↑myoglobin × 1.8	↑CK × 3.8 normal myoglobin & LDH	Normal CK Normal LDH	Normal CK Normal LDH	Normal CK ↑LDH × 1.15
Outcome	Listed for HTx at 3 y.o	Death at 2 years of age	Death at 9 years of age		HTx at 18 y.o		Listed for HTx at 12 y.o	ICD				

*AF* atrial fibrillation, *ASD* atrial septal defect, *AVBI* atrio-ventricular block I, *CoA* coarctation of the aorta, *CK* creatine kinase, *CK-MB* creatine kinase myocardial band, *CNS* central nervous system, *ECG* electrocardiography, *ENMG* electroneuromyography, *F* female, *HCM* hypertrophic cardiomyopathy, *HTx* heart transplantation, *LDH* Lactate dehydrogenase, *LV* left ventricle, *M* male, *MRI* magnetic resonance imaging, *RBBB* right bundle branch block, *RCM* restrictive cardiomyopathy, *ICD* Implantable Cardioverter Defibrillator, *SCD* sudden cardiac death, *SVT* supraventricular tachycardia, *VSD* ventricle septal defect, *VT* ventricular tachycardia

### Cardiac phenotype

**Fig. 1:**
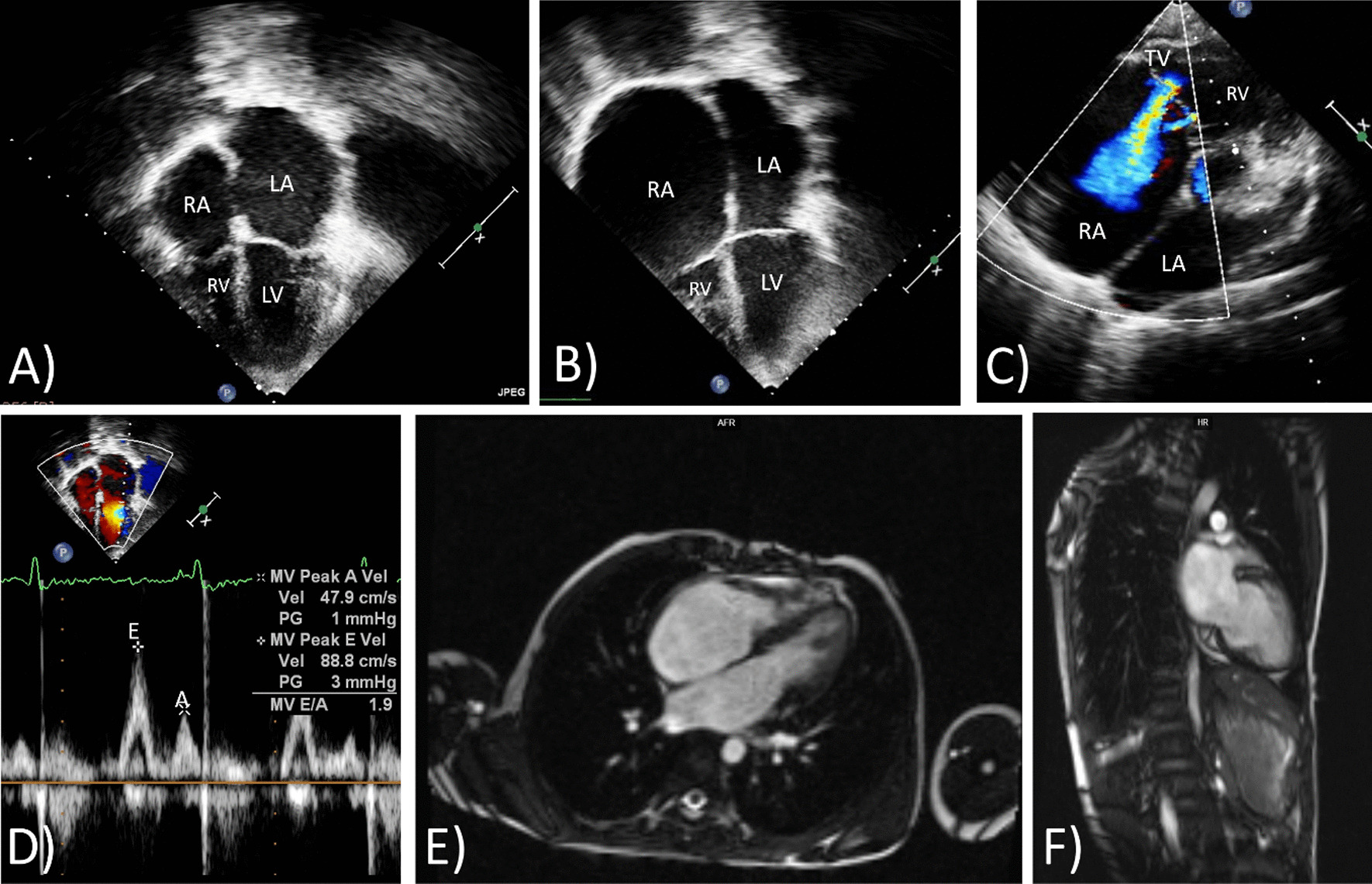
Cardiac imaging features of restrictive cardiomyopathy in Patient 7 (**b, c, e, f**) and Patient 10 (**a, d**). Images of patient 10 corresponding to four-chamber view (**a**) and Pulsed Way Doppler on mitral valve (**d**) confirmed atrial enlargement and moderate left ventricle diastolic dysfunction. Echocardiography pictures of patient 7 (**b, c**) corresponding to four-chamber view (**b**) and short-axis view with Color Doppler (**c**) also confirmed atrial enlargement with normal ventricle wall thickness and severe tricuspid valve regurgitation. Cardiac MRI images of patient 7 (**e, f**) demonstrating atrial enlargement and normal ventricle wall thickness. *RA* right atrium, *LA* left atrium, *RV* right ventricle, *LV* left ventricle, *TV* tricuspid valve

The cardiac phenotype was represented by RCM in all patients (100%), often in combination with septal defects such as atrial septal defect (4 out of 12; 33%) and ventricular septal defects (4 out of 12; 33%). In one case, there was a coexistent aortic arch hypoplasia. An increase in ventricular wall thickness was observed in ten patients (the mean Z-score of the interventricular septum was 4. Additional file 2), one child had signs of left ventricular non-compaction (LVNC), and one patient had signs of HCM (this patient was included since a transformation of the HCM phenotype into the RCMP was observed later on). Atrial enlargement was registered in all patients according to restrictive phenotype with the mean Z-score of 5.29 (Fig. 1a–d). Atrioventricular (AV) conduction defects were observed in three of twelve (25%) patients and were represented by first-degree AV block. In nine patients (75%), right bundle branch block was registered. Cardiac MRI with delayed post-contrast images was performed in four children and showed no clear pathological contrast delay in two patients (Patients 3 and 7, Fig. 1e, f) and minimally pronounced intramural fibrous changes in the basal or mid-apical parts of the anteroseptal region in Patient 10 and 4.

The average age of cardiac symptoms onset was 2.8 years. The most common symptoms at presentation were delay in weight and height gain and dyspnea. In five children, the diagnosis was suspected at a preclinical stage due to echocardiographic screening in the neonatal period and at 6 months of age. These children had concomitant congenital heart defects as identified by echocardiography, i.e. atrial or ventricular septal defects. None of the children had episodes of life-threatening arrhythmias, sustained ventricular tachycardia, non-sustained ventricular tachycardia, or experienced sudden cardiac death. Atrial fibrillation was registered in 1 patient, and none of the patients experienced a stroke or transient ischemic episodes. Among cardiac enzymes, CK-MB was often elevated 1.5–4 times the upper limit of the normal range while Troponin elevation was detected in only one patient out of nine (not available in 3). An increase in the level of NT-pro BNP was observed in all patients if available, on average 27 times. Heart failure symptoms were present in all patients, and in five (41.7%) was the first symptom at disease. One patient underwent successful heart transplantation at the age of 18 years. One patient had ICD installed as primary prevention of SCD. Two patients are currently on the waiting list for heart transplantation, and two died due to congestive heart failure.

### Neuromuscular phenotype

**Fig. 2:**
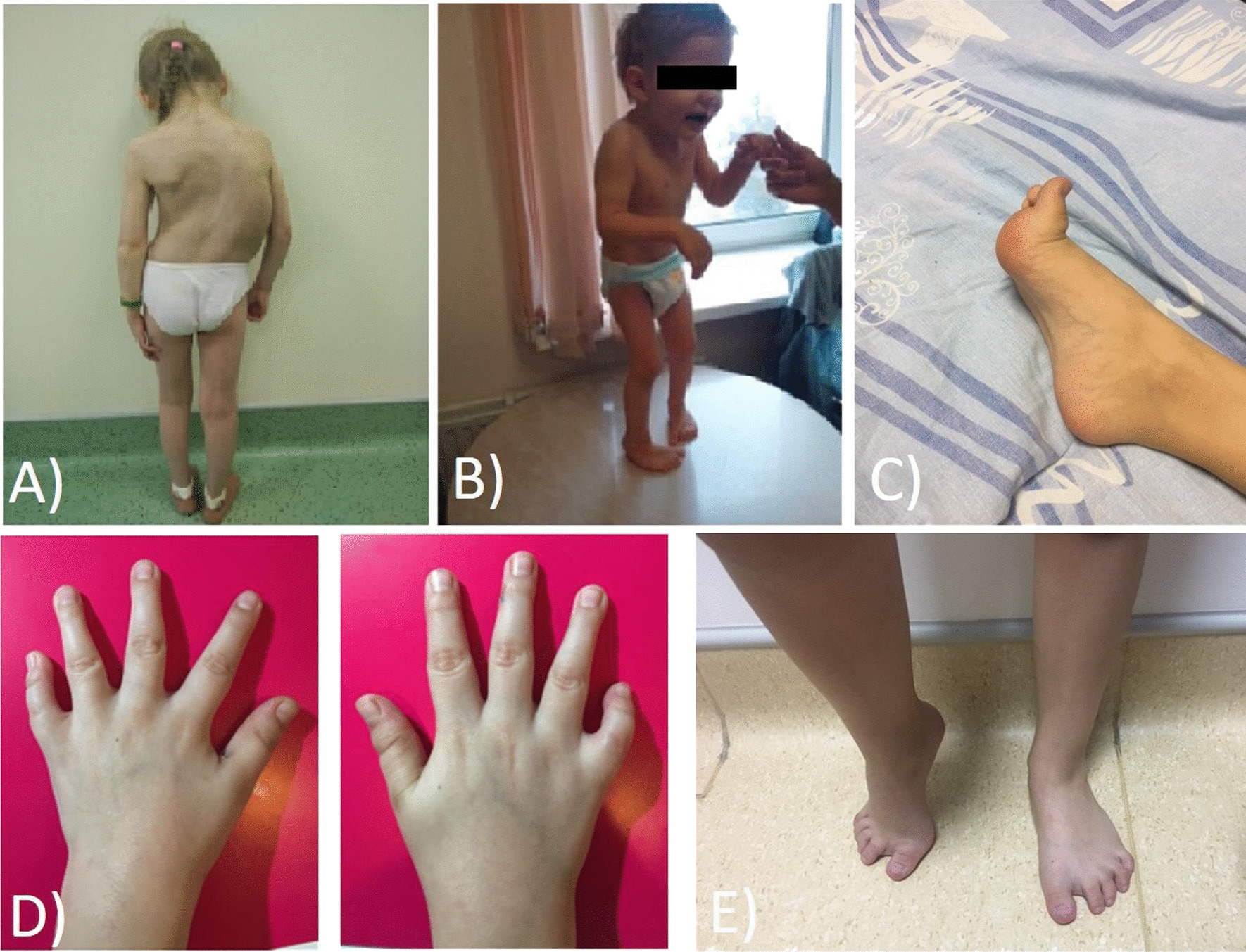
Clinical manifestations of *FLNC* filaminopathy. **a** Patient no. 3, deformity of the chest and spine (grade IV scoliosis). **b** Patient no. 2, signs of central tetraparesis, spasticity with myoclonus of the arms and legs, kyphoscoliosis. **c, e** Patient no. 12, arthrogryposis with joint deformity. **d** Patient no. 6, camptodactyly of the thumbs on both hands (reproduced from Vershinina et al. Pediatria 2020, 10.24110/0031-403X-2020-99-3-88-95 [63] permission applied)

In most of the cases, the neuromuscular phenotype became evident at birth or during the first year of life. The leading symptoms of neuromuscular phenotype in most patients were skeletal muscle hypotension and weakness, mainly with a limb-girdle pattern. In some patients, distal myopathy (DM) was also present, leading to gait difficulty including toewalking. Foot drop was, however, not observed in any of the patients. One-third of the patients demonstrated facial weakness and ptosis (5 out of 12; 41.7%), half also had scoliosis (6 out of 12; 50%), two patients developed severe progressive scoliosis, and seven had weakness of scapular muscle (7 out of 12; 58.3%) (Fig. 2a, b). Respiratory muscle involvement was not observed in any of the patients. Umbilical and *linea alba hernias* were also commonly reported (5 out of 12; 41.6%). Five out of twelve patients reported hip dysplasia at birth. In six patients, arthrogryposis was found at birth and remained clinically significant with age in three of them, leading to foot deformities and gait abnormalities (Fig. 2c, e). Another common symptom was *torticollis* (3 out of 12; 25%), leading to surgical correction in one patient. In two of these three patients, a very typical hand phenotype was observed presenting as camptodactyly of digits IV-V of the hand (Fig. 2d). Of note, most of the patients also had short stature and microsomia (5 out of 12, 41.6%).

Clinical signs of peripheral nervous system involvement were detected in one patient. Myopathy signs were registered by electromyography (EMG) in five patients, and neuropathy signs were also detected in three patients, overlapping with myopathic changes in one. In two patients, pyramidal signs were observed, combined with clonic seizures in one of them. Mild elevation of skeletal muscle enzymes (creatine kinase (CK), lactate dehydrogenase) was noted in half of the patients but did not exceed 1.5–2 times of the upper normal limit except for patient 4 (↑CK × 4).

Morphological examination was performed on tissue samples from only two patients (Patients 2 and 5). A post-mortem light microscopy analysis of skeletal and cardiac tissue was performed for patient 2 and did not reveal any intracellular aggregates or other signs of MFM, but confirmed extensive cardiac fibrosis and Z-line streaming, previously reported by Kiselev et al. [24]. In patient 5, muscle biopsy analysis revealed centrally located nuclei and signs similar to cytoplasmic body myopathy.

### Genetics and family history

**Fig. 3:**
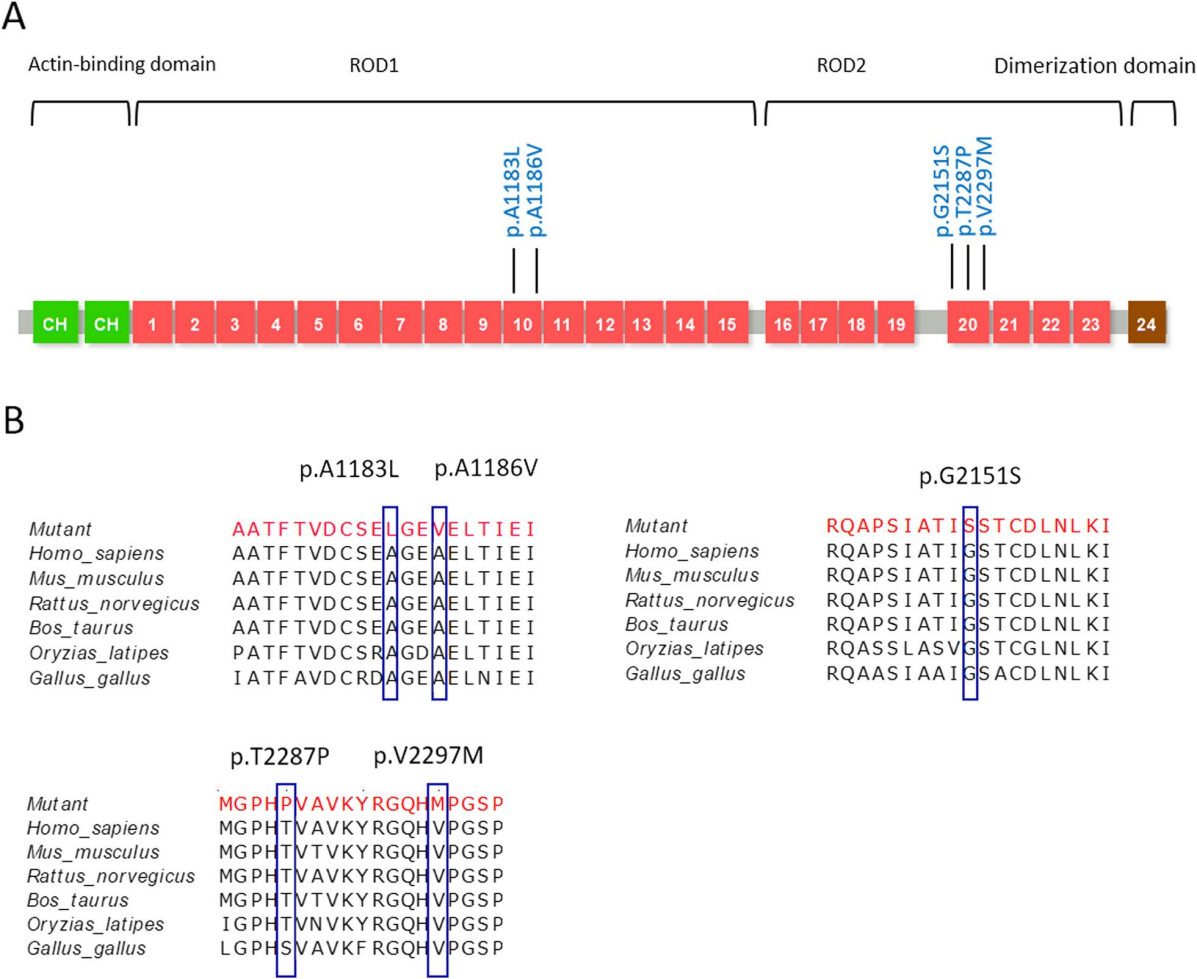
**a** Mutation plot and domain organization of the *FLNC* protein sequence performed using the UniProt database. *CH* calponin homology domain is marked with green; the ROD1 and ROD2 domains are marked with red, Ig-like domains are numbered from 1 to 24; the dimerization domain is marked with brown. Variants are mapped to the protein structure. **b** All missense variants were located in conserved regions among different species

In ten out of twelve patients, the disease was associated with missense variants of *FLNC*, and only in two cases (Patients 6 and 10), *FLNC* LOF variants were detected. Of note, in half of the cases, the phenotype was associated with Ala1186Val amino acid substitution and in one case with closely located A1183L variant. According to American College of Medical Genetics, most variants were classified as pathogenic or likely-pathogenic. The distribution of the variants is schematically illustrated on Fig. 3a. Most commonly, the missense variants fall into the ROD1 domain, of which six A1186V variants and one A1183L variant are in Ig‐like domain 10 (IgFLNc10), a well-known hotspot for myopathy phenotype [55]. An additional splice site mutation in the ROD1 domain is located in IgFLNc15, a site also reported in connection to myopathic phenotype [62]. The other four variants fall into the ROD2 domain, residing in the IgFLNc20-23. Three have been reported previously among the seven described variants, and four are newly described. The 4 bp deletion involving the splice site of exon 28 and located in IgFLNc15 has never been reported before. However, a mutation involving the same splice site c.4927+2T>A has been previously published in a patient with the myopathic phenotype [62]. No variants were detected in the actin-binding domain or dimerization domain of the *FLNC* gene. In six out of twelve patients, the parents were available for genotyping, and in five out of six cases, the mutation status was confirmed as de novo and in one case (Patient 7), the mutation was present in the mother. Her examination at the age of 49 revealed restrictive cardiac phenotype detected by echocardiography (left and right atrial enlargement—105 ml and 56 ml respectively, minimal left ventricular myocardial hypertrophy, signs of grade 3 diastolic dysfunction), permanent atrial fibrillation and surgically corrected cervix dystopia in childhood.

### Structural analysis of FLNC variants: homology modelling and structure comparison

Protein variants Ala1183Leu, Ala1186Val, Gly2151Ser, Thr2287Pro, Val2297Met, and Gly2569Ser of filamin C are found in highly conserved positions among different species (Fig. 3b). All of them are localized in immunoglobulin-like domains (Fig. 3a) which consist of seven antiparallel β-strands forming a barrel-like shape. The Ig-like domain is probably the most widespread protein module [49] and is involved in a variety of functions, including cell–cell recognition, cell-surface receptors, muscle structure, and the immune system.

**Fig. 4:**
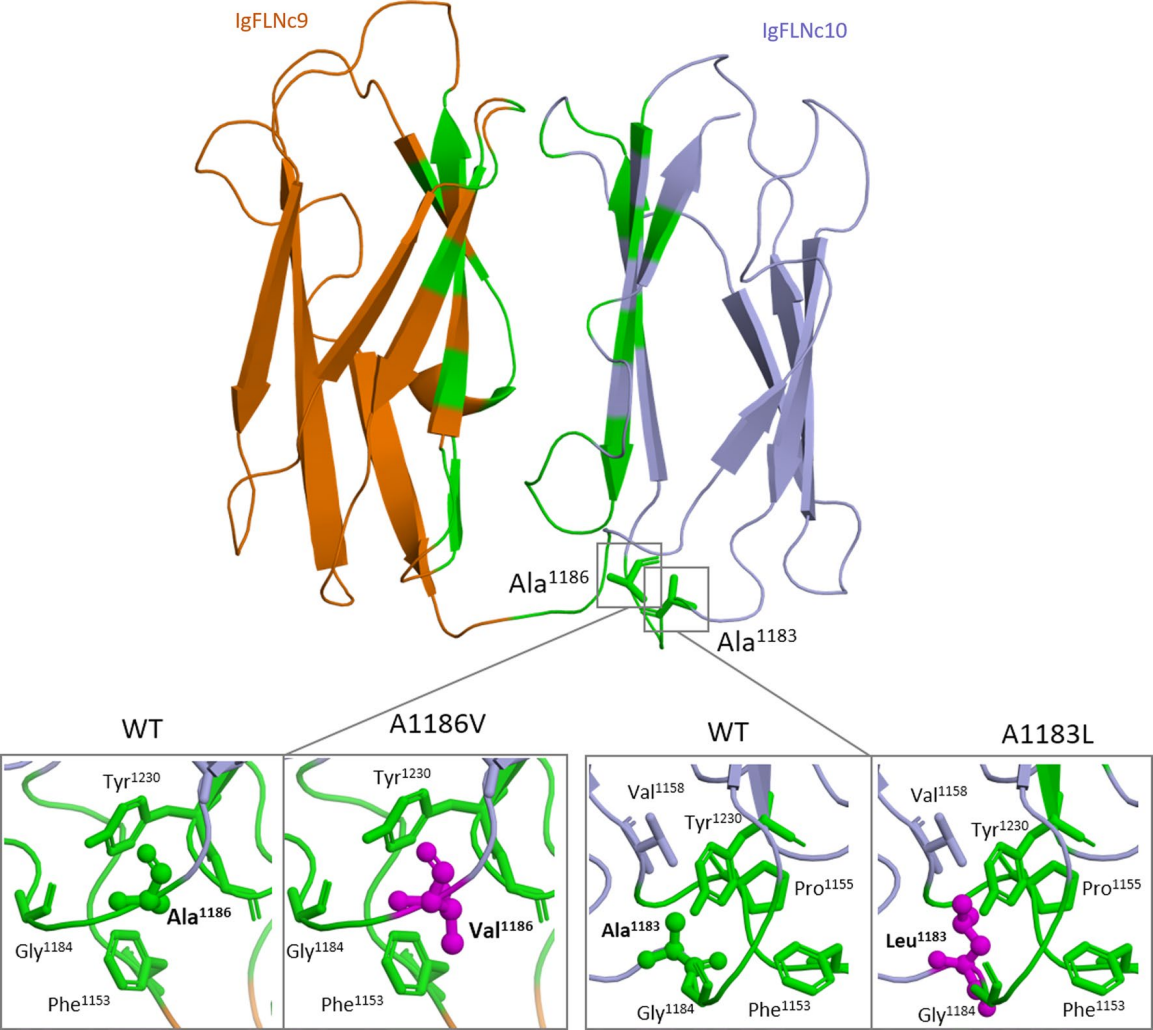
Views of the homology model of domain pairs of IgFLNc9–IgFLNc10, where domains 9 and 10 are shown as cartoons and colored in orange and light blue, respectively. The domain-domain interfaces are highlighted with green. Residues are shown as sticks. A1183 and A1186 are shown as ball and sticks; mutations A1186V and A1183L are colored with magenta

Variants p.Ala1183Leu and p.Ala1186Val are both localized in IgFLNC10 domain (Figs. 3a, 4). Residues of alanine in positions 1183 and 1186 fell in the inter-strands loop between B and C strands, which also connects two beta sheets formed by strands A–B–E–D and C–F–G and participate in IgFLNc9-IgFLNc10 domain-domain interface formation (Fig. 4). According to the structure-based prediction method SDM substitution p.Ala1183Leu has destabilizing effects on domain complex structure (Table 2). Substitutions in this region can lead to changes in the domain–domain interface and have a negative impact on filamin C stretching.

**Table 2:** Amino-acid substitutions in immunoglobulin-like domains

Substitution	Domain coordinates^a^	Ig^b^	*C*_*s*_^*c*^	PDB^d^	Identity^e^ (%)	RSA^f^ (%)	SDM^g^
A1183L	1150–1244	10	10	4m9p	73.1	0.0	D
A1186V	1150–1244	10	10	4m9p	73.1	21.5	S
G2151S	2129–2306	20	10	2j3s	62.4	40.1	D
T2287P	2129–2306	20	8	2j3s	62.4	39.4	D
V2297M	2129–2306	20	10	2j3s	62.4	1.0	D

The table shows data for filamin C (FLNC)

^a^Sequence positions (start and end) of the domain containing the substitution

^b^Ig domain number in a protein

^c^Conservation score of a mutated position in the alignment

^d^PDB ID of the template used for homology modeling of domain 3D structure

^e^Percent identity between the query and the PDB sequence

^f^Relative solvent-accessible area of the mutated residue, calculated by SDM

^g^Tool to predict changes in protein stability upon point mutations; ‘S’: mutation increases protein stability; ‘D’: mutation decreases protein stability

**Fig. 5:**
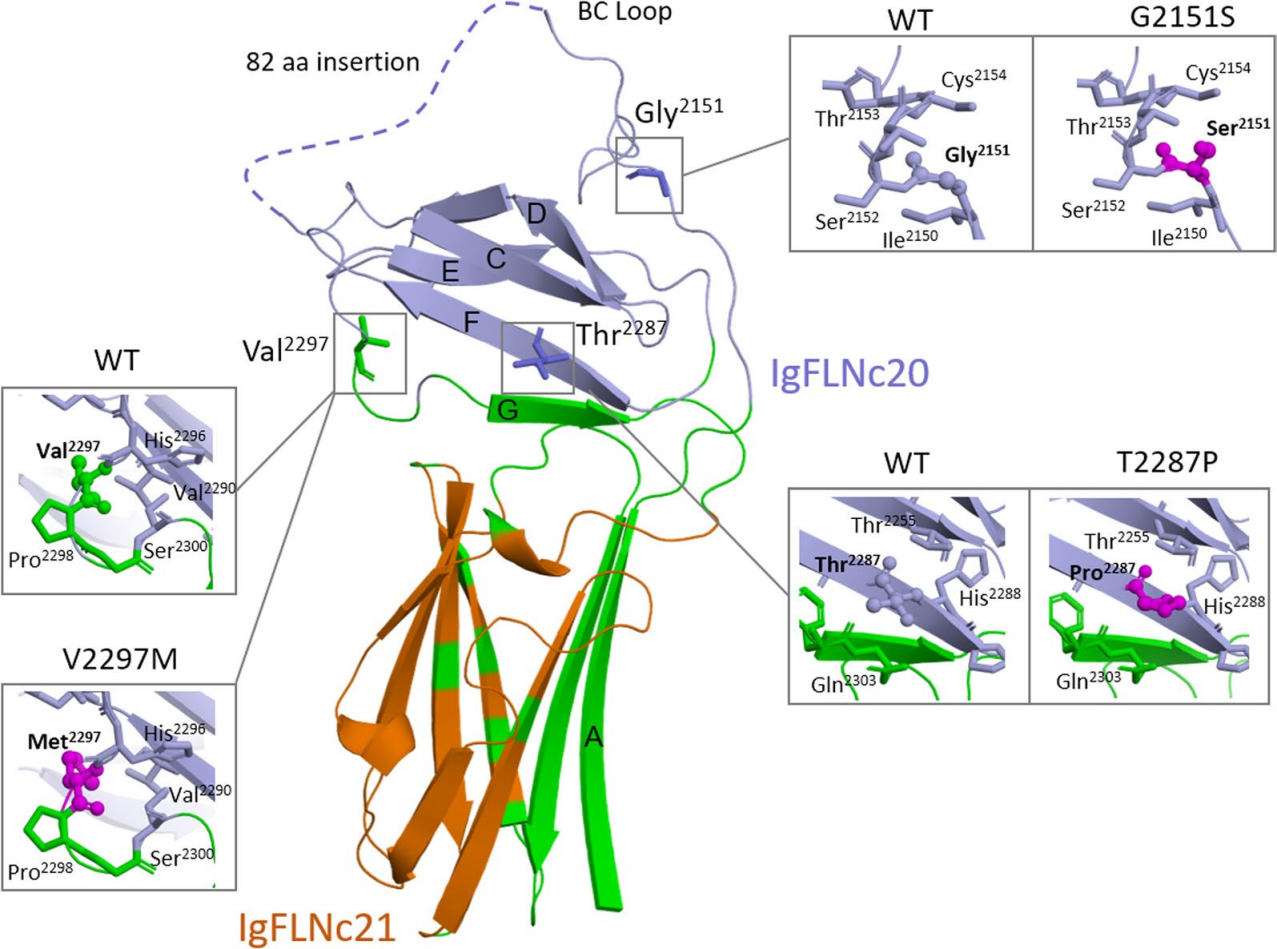
Views of the homology model of IgFLNc20-IgFLNc21, where domains 20 and 21 are shown as cartoons and colored in orange and lightblue, respectively. The domain-domain interface are highlighted in green. Residues are shown as sticks. V2297, G2151, and T2287 are shown as ball and sticks; mutations V2297M, T2287P, G2151S are colored with magenta. Note that the CD face of IgFLNc21 is blocked by strand A of IgFLNc20. An insertion of 82 amino acids in IgFLNc20 is shown by dashed line. It is a specific insertion which is absent in filamin A and filamin B

Variant p.Gly2151Ser is found in IgFLNc20 domain (Figs. 3a, 5). We analyzed a structure of FLNA fragment [28], IgFLNa19-21, which is very similar to IgFLNc19-21 fragment. In this structure, Ig20 is partially unfolded and its first part is separated from the rest of Ig20 and forms A strand next to the integrin-binding CD face of Ig21. Ig domains interact with other proteins by a beta-sheet augmentation mechanism. In this mechanism, the interaction partner forms an additional beta-strand next to the strand C of the filamin domain and simultaneously interacts with the hydrophobic groove between the strands C and D, called the CD face [23]. Disruption of this interaction enhances filamin binding to integrin beta-tails [28]. As seen in Fig. 5, p.Gly2151Ser is localized in an unfolded region between A and B strands of IgFLNc20 which participates in regulation of integrin-binding site.

Variant p.Thr2287Pro is localized on the F beta-strand of IgFLNc20. Replacement of Thr by Pro in beta strand may lead to unfolding and domain destabilization.

Domain IgFLNc20 lies on top of IgFLNc21, interacting mainly with BC loop of IgFLNc21. Variant p.Val2297Met is localized in the inter-strands loop between F and G strands (FG loop) of IgFLNc20 which participate in IgFLNc20-IgFLNc21 domain-domain interface formation (Fig. 5).

According to the structure-based method (SDM) variants p.Gly2151Ser, p.Thr2287Pro, and p.Val2297Met reduce the stability of Ig domains (Table 2).

Our findings indicate that pathogenic mutations are likely to disrupt interdomain interfaces, interfere with protein interactions, and affect protein stability, potentially destabilizing the multi-domain architecture of Ig domains.

## Discussion

Extensive genetic research of cardiac and neuromuscular disorders brought attention to the *FLNC* gene due to the growing evidence of its role in a variety of pathologies affecting cardiomyocytes and striated skeletal muscle cells. Following the initial description of *FLNC* association with MFM of limb-girdle type [56], several neuromuscular diseases were linked with this gene [17, 25]. In most cases, *FLNC* mutations lead to MFM due to alteration in the dimerization and ROD domains. However, in rare cases, mutations result in DM with no signs of MFM and intracellular protein aggregates, e.g., mutations in the actin-binding domain [15]. Later, *FLNC* variants were reported as a cause of isolated cardiac disorders, first for HCM and then for DCM, RCM, and ACM. Nowadays, after additional reports on *FLNC* mutations in patients with LVNC, hypoplastic heart syndrome, or arrhythmogenic mitral valve pathology, this gene is deemed to be a significant candidate for any type of inherited cardiac disorder, including arrhythmogenic phenotype with structurally normal heart [3, 4, 20, 51].

Even though cardiac pathologies were occasionally reported in *FLNC*-associated MFM or DM [25], the combined and clinically significant involvement of both cardiac and skeletal muscle systems are not very common, especially at an early age [1, 38, 55]. In addition, only a few cases of filaminopathies presenting in childhood have hitherto been reported, and then mainly with cardiomyopathy phenotype [8, 44, 61]. Recently we presented four cases of such rare combination of cardiac and muscular phenotype with presentation in early childhood [24]. The current study includes additional eight patients with more detailed clinical phenotyping and follow up as well as structural analysis of the causative variants. Therefore, the current report, describing twelve cases of early-onset RCM with congenital myopathy of predominantly limb-girdle pattern, underlines a distinct unique phenotype that can be suggested as a separate clinical form of filaminopathies.

The most typical feature found in all described cases was an early onset of the disease symptoms despite the *FLNC* variants being heterozygous. In previously reported cases of DM and MFM, the disease manifestation mainly occurred later in life, with the first symptoms becoming evident in the third-fifth decades of life [25, 26]. The only exception is the congenital form of myopathy caused by a homozygous *FLNC* mutation and characterized by generalized muscle weakness and the absence of cardiomyopathy signs [27]. In most cases of *FLNC-*associated RCM, clinical manifestation occurred in adulthood and was not combined with overt MFM or DM [33], 52]. Several pediatric RCM and HCM cases were reported with no signs of neuromuscular symptoms [8, 43]. The most similar cases to ours were reported by Xiao, who described two patients with early-onset RCM due to *FLNC* mutations [61]. While no detailed neuromuscular phenotype was described in the study, early-onset RCM caused by A1186V makes this case very similar to the cases from our study.

Importantly, no ventricular arrhythmias were noted in any of the patients presented here. DCM and ACM associated with causative *FLNC* variants are known also to be associated with increased risk of life-threatening arrhythmic events, independently of ejection fraction and structural remodelling [2, 20, 33, 42]. Currently, it is not clear if the increased arrhythmic risk is an attribute of only dilated/arrhythmogenic phenotype due to LOF *FLNC* variants, and it is also not clear if it can be observed only in groups of adult patients. Of note, we did not register any ventricular arrhythmias even in two patients with LOF *FLNC* variants (Patients 6 and 10). Since no cardiac MRI was performed, it is not possible to judge whether the absence of severe arrhythmic phenotype is explained by low degree/absence of myocardial fibrosis or by other reasons, for example, by distinct molecular mechanisms of *FLNC*-associated RCM compared to DCM and ACM. Despite the restrictive phenotype and marked increase of left atrium dimensions in most patients, no episodes of atrial fibrillation were found. Therefore, the observed phenotype of early-onset RCM was neither associated with ventricular arrhythmias nor with atrial fibrillation and flutter in childhood age. Of note, the described restrictive phenotype can potentially be associated with atrial fibrillation in the adult age as observed in the mother of Patient 7. However, it was not often seen in childhood and upon cardiomyopathy presentation. In two deceased patients, death occurred due to progressive heart failure, while three other patients required heart transplantation. Thus, progressive heart failure represented one of the leading causes of reaching clinical endpoints in half of the described cases, and implied a bad prognosis of pediatric RCM in general [41, 60].

The myopathic phenotype described here has several typical features. In most patients, the myopathic features were noted from birth and presented as muscular hypotension and weakness of predominantly proximal muscles. Distal muscle involvement was evident only in three patients, and respiratory weakness was not manifested in any children. Since morphological confirmation of MFM was possible only for one patient, the existence of true myofibrillar aggregates as a cause of limb-girdle myopathy in all described cases remains questionable. However, the possible combination of limb-girdle dystrophy and MFM myopathy and even different neuromuscular phenotypes due to the same mutations have been confirmed previously in filaminopathies by other authors [9, 19, 39, 54]. Despite the myopathic phenotype, no marked but only slight CK elevation was noted, indicating no or very mild dystrophic process involved. Further, myopathic changes were confirmed by EMG only in some of the patients. Such discrepancy between clinical symptoms, EMG, and biochemical data has been previously observed in filaminopathies and reported by other groups [1, 54]. Other notable features observed in several patients were: microsomia, hip dysplasia, congenital hernia, as well as a very remarkable limb phenotype. To the best of our knowledge, a combination of RCM, scoliosis, camptodactyly, torticollis, and arthrogryposis have not been previously reported in other patients with *FLNC* mutations and, being present in almost half of the studied patients, are suggested to represent typical features of the described pediatric form of filaminopathy. Previously, another genetic basis of camptodactyly was reported by Deng et al. [13], who linked this hand phenotype to mutations in *TLN2* gene encoding the talin 2. Similar to filamin C, this protein participates in cross-linking the cytoplasmic domains of integrin subunits to actin filaments [10]. Scoliosis and flexion contracture of the thumb were also described in the *PYROXD1*-linked form of MFM [32]. Similarly, several genetic causes of congenital arthrogryposis and torticollis have also been identified [11, 14, 21, 31, 57], underscoring the inherited etiology of these conditions. Thus, the described manifestation of myopathy and contractures can be seen in the frame of filaminopathy phenotype. In some patients, cardiac and skeletal muscle features were combined with the involvement of the central and peripheral nervous systems, mainly presenting as signs from the pyramid tract. Together with the cases reported by Tasca et al. and Previtali et al., this underlines the importance of filamin C in neuronal cells despite the low expression level and the existence of other filamins, namely filamin A and B [35, 50].

Our study has several important limitations. Firstly, parental genotyping was not performed for all the patients. Therefore, the de novo status of mutation was confirmed only for five out of twelve patients. Another limitation is a lack of extensive morphological analysis of muscle tissue and myocardium due to technical and ethical limitations in obtaining a muscular biopsy from pediatric patients. Thus, the presence of MFM and protein aggregates remains unknown for most cases. Similarly, cardiac and skeletal muscle MRI has not been performed, and hence the exact degree of myocardial fibrosis and muscle wasting is challenging to estimate.

## Conclusions

We summarized the clinical information and typical features of early-onset RCM associated with congenital myopathy, arthrogryposis, camptodactyly, and torticollis due to *FLNC* mutations in twelve pediatric patients. In half of the described cases, an amino acid substitution A1186V, altering the structure of IgFLNc10, was found. This mutation defines a mutational hotspot for the reported combination of myopathy and cardiomyopathy. Presentation of this combination becomes evident early in childhood, and it is not commonly observed in filaminopathies that, with rare exceptions, are considered as isolated cardiac or muscular pathologies. Early disease presentation and unfavorable prognosis of heart failure demanding heart transplantation make awareness of this clinical form of filaminopathy of great clinical importance for pediatric cardiologists and neurologists.

## Supplementary Information

**Additional file 1.**. List of studied genes

**Additional file 2. Table S1**. Echocardiography features of patients with FLNC-associated restrictive cardiomyopathy.

## Data Availability

The analyzed datasets for this study can be found in at SRA database under the reference number SRR16609854.

## Abbreviations

*FLNC*: Filamin-C
*FLNA*: Filamin-A
*TTN*: Titin
LOF: Loss of function
RNA: Ribonucleic acid
RCM: Restrictive cardiomyopathy
HCM: Hypertrophic cardiomyopathy
ACM: Arrhythmogenic cardiomyopathy
DCM: Dilated cardiomyopathy
MFM: Myofibrillar myopathy
DM: Distal myopathy
EMG: Electromyography
SCD: Sudden Cardiac Death
ICD: Implantable Cardioverter Defibrillator
MRI: Magnetic resonance imaging
SDM: Site Directed Mutator

## References

[1] Ader F (2019). FLNC pathogenic variants in patients with cardiomyopathies: prevalence and genotype-phenotype correlations. Clin Genet.

[2] Akhtar MM (2021). Association of left ventricular systolic dysfunction among carriers of truncating variants in Filamin C with frequent ventricular arrhythmia and end-stage heart failure. JAMA Cardiol.

[3] Avila-Smirnow D (2016). Cardiac arrhythmia and late-onset muscle weakness caused by a myofibrillar myopathy with unusual histopathological features due to a novel missense mutation in FLNC. Rev Neurol (Paris).

[4] Bains S (2019). A novel truncating variant in FLNC-encoded Filamin C may serve as a proarrhythmic genetic substrate for arrhythmogenic bileaflet mitral valve prolapse syndrome. Mayo Clin Proc.

[5] Batonnet-Pichon S (2017). Myofibrillar myopathies: new perspectives from animal models to potential therapeutic approaches. J Neuromuscul Dis.

[6] Begay RL (2018). Filamin C truncation mutations are associated with arrhythmogenic dilated cardiomyopathy and changes in the cell-cell adhesion structures. JACC Clin Electrophysiol.

[7] Begay RL (2016). FLNC gene splice mutations cause dilated cardiomyopathy. JACC Basic Transl Sci.

[8] Brodehl A (2016). Mutations in FLNC are associated with familial restrictive cardiomyopathy. Hum Mutat.

[9] Carvalho AAS (2018). Genetic mutations and demographic, clinical, and morphological aspects of myofibrillar myopathy in a French cohort. Genet Test Mol Biomarkers.

[10] Critchley DR (2008). Talin at a glance. J Cell Sci.

[11] Culic V (2016). Distal arthrogryposis with variable clinical expression caused by TNNI2 mutation. Hum Genome Var.

[12] Delort F (2019). Alterations of redox dynamics and desmin post-translational modifications in skeletal muscle models of desminopathies. Exp Cell Res.

[13] Deng H (2016). Exome sequencing of a pedigree reveals S339L mutation in the TLN2 gene as a cause of fifth finger camptodactyly. PLoS ONE.

[14] Duan BC (2019). Alternating hemiplegia and paroxysmal torticollis caused by SCN4A mutation: a new phenotype?. Neurology.

[15] Duff RM (2011). Mutations in the N-terminal actin-binding domain of filamin C cause a distal myopathy. Am J Hum Genet.

[16] Eden M (2021). Cardiac filaminopathies: illuminating the divergent role of Filamin C mutations in human cardiomyopathy. J Clin Med.

[17] Evangelista T (2020). A heterozygous mutation in the Filamin C gene causes an unusual nemaline myopathy with ring fibers. J Neuropathol Exp Neurol.

[18] Furst DO (2013). ilamin C-related myopathies: pathology and mechanisms. Acta Neuropathol.

[19] Guergueltcheva V (2011). Distal myopathy with upper limb predominance caused by filamin C haploinsufficiency. Neurology.

[20] Hall CL (2020). Filamin C variants are associated with a distinctive clinical and immunohistochemical arrhythmogenic cardiomyopathy phenotype. Int J Cardiol.

[21] Jin JY (2020). The novel compound heterozygous mutations of ECEL1 identified in a family with distal arthrogryposis type 5D. Biomed Res Int.

[22] Jorholt J (2020). Two new cases of hypertrophic cardiomyopathy and skeletal muscle features associated with ALPK3 homozygous and compound heterozygous variants. Genes (Basel).

[23] Kiema T (2006). The molecular basis of filamin binding to integrins and competition with talin. Mol Cell.

[24] Kiselev A (2018). De novo mutations in FLNC leading to early-onset restrictive cardiomyopathy and congenital myopathy. Hum Mutat.

[25] Kley RA (2007). Clinical and morphological phenotype of the filamin myopathy: a study of 31 German patients. Brain.

[26] Kley RA (2021). FLNC-associated myofibrillar myopathy: new clinical, functional, and proteomic data. Neurol Genet.

[27] Kolbel H (2020). First clinical and myopathological description of a myofibrillar myopathy with congenital onset and homozygous mutation in FLNC. Hum Mutat.

[28] Lad Y (2007). Structure of three tandem filamin domains reveals auto-inhibition of ligand binding. EMBO J.

[29] Luan X (2010). A novel heterozygous deletion-insertion mutation (2695–2712 del/GTTTGT ins) in exon 18 of the filamin C gene causes filaminopathy in a large Chinese family. Neuromuscul Disord.

[30] Mao Z (2020). Structure and function of Filamin C in the muscle Z-disc. Int J Mol Sci.

[31] Miltgen M (2016). Novel heterozygous mutation in ANO3 responsible for craniocervical dystonia. Mov Disord.

[32] O'Grady GL (2016). Variants in the oxidoreductase PYROXD1 cause early-onset myopathy with internalized nuclei and myofibrillar disorganization. Am J Hum Genet.

[33] Ortiz-Genga MF (2016). Truncating FLNC mutations are associated with high-risk dilated and arrhythmogenic cardiomyopathies. J Am Coll Cardiol.

[34] Pires DEV (2014). DUET: a server for predicting effects of mutations on protein stability using an integrated computational approach. Nucleic Acids Res.

[35] Previtali SC (2019). Expanding the central nervous system disease spectrum associated with FLNC mutation. Muscle Nerve.

[36] Richards S (2015). Standards and guidelines for the interpretation of sequence variants: a joint consensus recommendation of the American College of Medical Genetics and Genomics and the Association for Molecular Pathology. Genet Med.

[37] Rojnueangnit K (2020). Identification of gene mutations in primary pediatric cardiomyopathy by whole exome sequencing. Pediatr Cardiol.

[38] Roldan-Sevilla A (2019). Missense mutations in the FLNC gene causing familial restrictive cardiomyopathy. Circ Genom Precis Med.

[39] Rossi D (2017). A novel FLNC frameshift and an OBSCN variant in a family with distal muscular dystrophy. PLoS ONE.

[40] Ruparelia AA (2021). Metformin rescues muscle function in BAG3 myofibrillar myopathy models. Autophagy.

[41] Russo LM (2005). Idiopathic restrictive cardiomyopathy in children. Heart.

[42] Sammani A (2020). Predicting sustained ventricular arrhythmias in dilated cardiomyopathy: a meta-analysis and systematic review. ESC Heart Fail.

[43] Schanzer A (2021). The p.Ala2430Val mutation in filamin C causes a "hypertrophic myofibrillar cardiomyopathy". J Muscle Res Cell Motil.

[44] Schubert J (2018). Novel pathogenic variants in filamin C identified in pediatric restrictive cardiomyopathy. Hum Mutat.

[45] Sethi R (2014). A novel structural unit in the N-terminal region of filamins. J Biol Chem.

[46] Shatunov A (2009). In-frame deletion in the seventh immunoglobulin-like repeat of filamin C in a family with myofibrillar myopathy. Eur J Hum Genet.

[47] Sievers F (2011). Fast, scalable generation of high-quality protein multiple sequence alignments using Clustal Omega. Mol Syst Biol.

[48] Sjekloća L (2007). Crystal structure of human Filamin C domain 23 and small angle scattering model for filamin C 23–24 dimer. J Mol Biol.

[49] Smith DK (1997). Sequence profiles of immunoglobulin and immunoglobulin-like domains. J Mol Biol.

[50] Tasca G (2012). Novel FLNC mutation in a patient with myofibrillar myopathy in combination with late-onset cerebellar ataxia. Muscle Nerve.

[51] Theis JL (2021). Genetic association between hypoplastic left heart syndrome and cardiomyopathies. Circ Genom Precis Med.

[52] Tucker NR (2017). Novel mutation in FLNC (Filamin C) causes familial restrictive cardiomyopathy. Circ Cardiovasc Genet.

[53] Valdes-Mas R (2014). Mutations in filamin C cause a new form of familial hypertrophic cardiomyopathy. Nat Commun.

[54] van den Bogaart FJ (2017). Widening the spectrum of filamin-C myopathy: predominantly proximal myopathy due to the p.A193T mutation in the actin-binding domain of FLNC. Neuromuscul Disord.

[55] Verdonschot JAJ (2020). A mutation update for the FLNC gene in myopathies and cardiomyopathies. Hum Mutat.

[56] Vorgerd M (2005). A mutation in the dimerization domain of filamin c causes a novel type of autosomal dominant myofibrillar myopathy. Am J Hum Genet.

[57] Wang WB (2020). Identification of a novel pathogenic mutation of the MYH3 gene in a family with distal arthrogryposis type 2B. Mol Med Rep.

[58] Waterhouse A (2018). SWISS-MODEL: homology modelling of protein structures and complexes. Nucleic Acids Res.

[59] Waterhouse AM (2009). Jalview Version 2—a multiple sequence alignment editor and analysis workbench. Bioinformatics.

[60] Webber SA (2012). Outcomes of restrictive cardiomyopathy in childhood and the influence of phenotype: a report from the Pediatric Cardiomyopathy Registry. Circulation.

[61] Xiao F (2020). Clinical exome sequencing revealed that FLNC variants contribute to the early diagnosis of cardiomyopathies in infant patients. Transl Pediatr.

[62] Zenagui R (2018). A reliable targeted next-generation sequencing strategy for diagnosis of myopathies and muscular dystrophies, especially for the giant titin and nebulin genes. J Mol Diagn.

[63] Vershinina T (2020). Features of the clinical phenotype of filamine cardiomyopathies with a debut in early childhood. Pediatria.

