# Supplementary material for: Rare clinical phenotype of filaminopathy presenting as restrictive cardiomyopathy and myopathy in childhood
Source: Orphanet J Rare Dis. 2022 Sep 14;17:358. doi: 10.1186/s13023-022-02477-5 (PMC9476594; doi:10.1186/s13023-022-02477-5)
Supplement: Supplementary file 1 — Additional file 1.. List of studied genes [file 13023_2022_2477_MOESM1_ESM.docx]

Supplementary Material

List of studied genes

|  | **Gene name** | **Interval (hg19)** |
| --- | --- | --- |
|  | ACADVL | chr17:7217136-7225107 |
|  | ACTA1 | chr1:229431489-229433125 |
|  | ACTC1 | chr15:34790402-34794818 |
|  | ACTN2 | chr1:236686664-236762629 |
|  | ACVR2B | chr3:38454313-38483342 |
|  | AGK | chr7:141555457-141652934 |
|  | AKAP9 | chr7:91941090-92110169 |
|  | ALPK3 | chr15:84816837-84868466 |
|  | ANK2 | chr4:112904484-113381650 |
|  | ANKRD1 | chr10:90912856-90921037 |
|  | ANO5 | chr11:22193483-22279775 |
|  | BAG3 | chr10:119651666-119677292 |
|  | BRAF | chr7:140726484-140924713 |
|  | CACNA1C | chr12:1971053-2691209 |
|  | CACNA2D1 | chr7:81950382-82443469 |
|  | CACNB2 | chr10:18140727-18539734 |
|  | CALM1 | chr14:90397221-90404727 |
|  | CALM2 | chr2:47160766-47176551 |
|  | CALM3 | chr19:46601425-46609163 |
|  | CALR3 | chr19:16479121-16496139 |
|  | CASQ2 | chr1:115701231-115768551 |
|  | CAV3 | chr3:8733867-8745877 |
|  | CBL | chr11:119206408-119308087 |
|  | CDH2 | chr18:27952143-28177032 |
|  | CMYA5 | chr5:79689898-79799626 |
|  | CRELD1 | chr3:9934429-9944589 |
|  | CRYAB | chr11:111908754-111911734 |
|  | CSRP3 | chr11:19182660-19192458 |
|  | CTNNA3 | chr10:65920320-67648780 |
|  | DES | chr2:219418453-219426000 |
|  | DMD | chrX:31121823-33339275 |
|  | DMPK | chr19:45770471-45782381 |
|  | DNAAF1 | chr16:84145431-84178416 |
|  | DNAAF3 | chr19:55159052-55166658 |
|  | DPP6 | chr7:153887674-154892490 |
|  | DSC2 | chr18:31068005-31101981 |
|  | DSG2 | chr18:31498242-31546753 |
|  | DSP | chr6:7541906-7585888 |
|  | DTNA | chr18:34755967-34890450 |
|  | DYSF | chr2:71453989-71686502 |
|  | EMD | chrX:154379475-154381207 |
|  | EYA4 | chr6:133274771-133531236 |
|  | FHL1 | chrX:136196793-136210035 |
|  | FHL2 | chr2:105361273-105399555 |
|  | FHOD3 | chr18:36297826-36779540 |
|  | FKRP | chr19:46755441-46756948 |
|  | FKTN | chr9:105575023-105640133 |
|  | FLNA | chrX:154348839-154371255 |
|  | FLNC | chr7:128830628-128858533 |
|  | FXN | chr9:69035773-69099944 |
|  | GAA | chr17:80104577-80119341 |
|  | GATA4 | chr8:11708303-11758485 |
|  | GATA5 | chr20:62464826-62475531 |
|  | GATA6 | chr18:22171135-22200833 |
|  | GATAD1 | chr7:92447720-92456572 |
|  | GDF1 | chr19:18868587-18870317 |
|  | GJA5 | chr1:147758152-147759248 |
|  | GLA | chrX:101397799-101407913 |
|  | GPD1L | chr3:32106702-32165920 |
|  | HAND1 | chr5:154475796-154478018 |
|  | HCN4 | chr15:73322471-73368280 |
|  | HFE | chr6:26087431-26094421 |
|  | HRAS | chr11:532626-534332 |
|  | ILK | chr11:6604262-6610621 |
|  | ISPD | chr7:16091685-16421332 |
|  | JPH2 | chr20:44114786-44186715 |
|  | JUP | chr17:41755734-41771864 |
|  | KCNA5 | chr12:5044138-5045999 |
|  | KCND3 | chr1:111776067-111982736 |
|  | KCNE1 | chr21:34449235-34449644 |
|  | KCNE2 | chr21:34370469-34370860 |
|  | KCNE3 | chr11:74457242-74457573 |
|  | KCNE5 | chrX:109624582-109625030 |
|  | KCNH2 | chr7:150945355-150977923 |
|  | KCNJ2 | chr17:70175030-70176333 |
|  | KCNJ5 | chr11:128911264-128916741 |
|  | KCNJ8 | chr12:21765713-21773626 |
|  | KCNQ1 | chr11:2444676-2848013 |
|  | KRAS | chr12:25209785-25245394 |
|  | LAMA4 | chr6:112109427-112254160 |
|  | LAMP2 | chrX:120428474-120469179 |
|  | LDB3 | chr10:86668682-86732986 |
|  | LEFTY2 | chr1:225937411-225941150 |
|  | LMNA | chr1:156114909-156139849 |
|  | LMOD3 | chr3:69109085-69122396 |
|  | LRRC10 | chr12:69609995-69610848 |
|  | LZTR1 | chr22:20982362-20997358 |
|  | MAP2K1 | chr15:66387338-66490625 |
|  | MAP2K2 | chr19:4090588-4123885 |
|  | MIB1 | chr18:21741574-21864676 |
|  | MMP21 | chr10:125766652-125775831 |
|  | MRAS | chr3:138372874-138402279 |
|  | MYBPC3 | chr11:47331861-47352657 |
|  | MYBPHL | chr1:109294229-109307001 |
|  | MYH6 | chr14:23382030-23407233 |
|  | MYH7 | chr14:23412844-23433742 |
|  | MYL2 | chr12:110911067-110920539 |
|  | MYL3 | chr3:46858234-46863400 |
|  | MYL4 | chr17:47209413-47223052 |
|  | MYLK2 | chr20:31819571-31833807 |
|  | MYOF | chr10:93306953-93482204 |
|  | MYOM1 | chr18:3067252-3215233 |
|  | MYOT | chr5:137870642-137887395 |
|  | MYOZ2 | chr4:119136516-119186210 |
|  | MYPN | chr10:68106715-68210465 |
|  | NEBL | chr10:20785737-21173843 |
|  | NEXN | chr1:77916097-77942923 |
|  | NF1 | chr17:31095198-31378941 |
|  | NKX2-5 | chr5:173232559-173235093 |
|  | NKX2-6 | chr8:23702441-23706608 |
|  | NPPA | chr1:11845993-11847694 |
|  | NRAS | chr1:114708525-114716170 |
|  | NUP155 | chr5:37291890-37370987 |
|  | PDLIM3 | chr4:185502284-185535444 |
|  | PKD1L1 | chr7:47775133-47948450 |
|  | PKP2 | chr12:32792414-32896741 |
|  | PLEC | chr8:143916167-143975379 |
|  | PLEKHM2 | chr1:15684549-15733944 |
|  | PLN | chr6:118558912-118559090 |
|  | PPA2 | chr4:105369715-105474060 |
|  | PPP1CB | chr2:28752115-28799313 |
|  | PRDM16 | chr1:3069250-3433821 |
|  | PRKAG2 | chr7:151557191-151876630 |
|  | PSEN1 | chr14:73148010-73219299 |
|  | PSEN2 | chr1:226880636-226895589 |
|  | PTPN11 | chr12:112419102-112504774 |
|  | RAF1 | chr3:12584504-12618731 |
|  | RANGRF | chr17:8288779-8289946 |
|  | RBM20 | chr10:110644445-110835988 |
|  | RIT1 | chr1:155900378-155910895 |
|  | RRAS | chr19:49635566-49640108 |
|  | RYR2 | chr1:237042512-237832657 |
|  | SALL4 | chr20:51784255-51802418 |
|  | SCN10A | chr3:38697339-38794020 |
|  | SCN1B | chr19:35030811-35039711 |
|  | SCN2B | chr11:118166877-118176441 |
|  | SCN3B | chr11:123634133-123653811 |
|  | SCN4B | chr11:118137017-118152683 |
|  | SCN5A | chr3:38550311-38633317 |
|  | SCNN1G | chr16:23186262-23215479 |
|  | SDHA | chr5:218346-256430 |
|  | SGCD | chr5:156329567-156759400 |
|  | SHOC2 | chr10:110964349-111011828 |
|  | SLMAP | chr3:57757642-57927398 |
|  | SNTA1 | chr20:33408497-33443630 |
|  | SOS1 | chr2:38985814-39120432 |
|  | SOS2 | chr14:50118334-50231293 |
|  | SPEG | chr2:219434968-219498199 |
|  | SPRED1 | chr15:38253176-38351674 |
|  | SYNE1 | chr6:152122426-152628341 |
|  | SYNM | chr15:99105190-99133068 |
|  | SYNPO2L | chr10:73646708-73655932 |
|  | TAZ | chrX:154411834-154421014 |
|  | TBX20 | chr7:35202420-35253630 |
|  | TBX5 | chr12:114355522-114403908 |
|  | TCAP | chr17:39665350-39666119 |
|  | TECRL | chr4:64277025-64409361 |
|  | TGFB3 | chr14:75959177-75980903 |
|  | TMEM43 | chr3:14125184-14141805 |
|  | TMPO | chr12:98515858-98547868 |
|  | TNNC1 | chr3:52451265-52454025 |
|  | TNNI3 | chr19:55151824-55157599 |
|  | TNNI3K | chr1:74235442-74543992 |
|  | TNNT2 | chr1:201359200-201373264 |
|  | TPM1 | chr15:63042820-63071182 |
|  | TRDN | chr6:123218591-123636785 |
|  | TRPM4 | chr19:49157857-49211508 |
|  | TTN | chr2:178527002-178804652 |
|  | TTR | chr18:31591893-31598685 |
|  | VCL | chr10:73998198-74118179 |
|  | ZIC3 | chrX:137566682-137577281 |
