# Supplementary material for: Rare clinical phenotype of filaminopathy presenting as restrictive cardiomyopathy and myopathy in childhood
Source: Orphanet J Rare Dis. 2022 Sep 14;17:358. doi: 10.1186/s13023-022-02477-5 (PMC9476594; doi:10.1186/s13023-022-02477-5)
Supplement: Supplementary file 2 — Additional file 2. Table S1. Echocardiography features of patients with FLNC-associated restrictive cardiomyopathy. [file 13023_2022_2477_MOESM2_ESM.docx]

Table S1. Echocardiography features of patients with *FLNC*-associated restrictive cardiomyopathy.

| Patient | Pt 1 | | Pt 2 | | Pt 3 | | Pt 4 | | Pt 5 | | Pt 6 | | Pt 7 | | Pt 8 | | Pt 9 | | Pt 10 | | Pt 11 | | Pt 12 | |
| --- | --- | --- | --- | --- | --- | --- | --- | --- | --- | --- | --- | --- | --- | --- | --- | --- | --- | --- | --- | --- | --- | --- | --- | --- |
| Age, years old | 10 |  | 2,5 |  | 9 |  | 8 |  | 15 |  | 9 |  | 14 |  | 8,5 |  | 2,5 |  | 8 |  | 9 |  | 6 |  |
| Weight, kg | 10,65 |  | 12 |  | 16,2 |  | 27,5 |  | 75 |  | 20 |  | 44,3 |  | 21 |  | 12 |  | 23 |  | 22 |  | 15,6 |  |
| Height, cm | 79,5 |  | 85 |  | 104 |  | 121,5 |  | 174 |  | 120 |  | 159 |  | 121 |  | 86 |  | 127,5 |  | 126 |  | 104 |  |
|  |  | z-score |  | z-score |  | z-score |  | z-score |  | z-score |  | z-score |  | z-score |  | z-score |  | z-score |  | z-score |  | z-score |  | z-score |
| IVS, mm | 10,3 | 7,63 | 6 | 2,12 | 6,4 | 2,13 | 9,8 | 4,67 | 12 | 4,18 | 6 | 1 | 9 | 2,56 | 10,3 | 6,38 | 6,5 | 2,75 | 7,5 | 2,11 | 7,5 | 2,88 | 12 | 9,6 |
| EDD, mm | 18,1 | -3,53 | 36,3 | 2,61 | 30 | -1,07 | 32,1 | -1,64 | 47 | 0,5 | 35,2 | -0,19 | 34,3 | -3 | 34,8 | -0,32 | 34 | 1,79 | 34 | -1,02 | 34,4 | -0,45 | 26,6 | -2,2 |
| ESD, mm | 12,9 | -2,05 | 23,4 | 2,7 | 19,5 | -0,38 | 13 | -3,79 | 29 | 0,13 | 22,3 | -0,16 | 20,3 | -2,61 | 22,9 | 0,08 | 21,4 | 1,70 | 18,5 | -1,82 | 20,8 | -0,76 | 15,8 | -1,96 |
| LVPWd, mm | 12 | 9,87 | 6 | 1,87 | 7 | 2,8 | 9,45 | 3,23 | 6 | -1,21 | 6 | 0,29 | 9 | 1,62 | 13,3 | 7,24 | 6,5 | 2,53 | 7,5 | 1,45 | 7,5 | 1,71 | 8 | 4,13 |
| EF, % (Simpson) | 53 |  | 65 |  | 70 |  | 67 |  | 60 |  | 56 |  | 64 |  | 64 |  | 66 |  | 65 |  | 56 |  | 68 |  |
| RV Mid-Cavitary Diameter, mm | 30,2 | 5,03 | 23,6 | 2,92 | 23,5 | 2,07 | 23 | 0,75 | 36 | 1,73 | 20 | 0,32 | 37,6 | 3,12 | 21 | 0,58 | 22,1 | 2,42 | 23 | 1,01 | 24 | 1,42 | 39 | 5,84 |
| LA volume, ml | 29 | 4,33 | 29 | 7,5 |  |  | 50 | 3,52 | 73 | 4,06 | 39 | 2,74 | 60 | 2,2 | 36 | 2,82 | 19 | 2,88 | 47 | 2,88 | 79 | 11,92 | 46 | 4,5 |
| LA area, cm2 | 13,1 | 6,18 | 19 | 7,93 | 18,8 | 6,56 | 18,7 | 3,19 | 19 | 4 | 13,9 | 3,81 | 20,6 | 3,27 | 22,8 | 6,65 | 12,8 | 5,51 | 18,2 | 4,91 | 19,4 | 5,45 | 16,9 | 6,02 |
| LA major diameter,mm | 43,6 | 5,95 | 56 | 7,99 | 47 | 5,2 | 52,8 | 4,8 | 66 | 3,96 | 24,5 | -1,94 | 74 | 6,5 | 52 | 5,3 | 44 | 5,60 | 46,3 | 3,86 | 51 | 4,93 | 48 | 5,5 |
| RA area, cm2 | 10,5 | 4,31 | 8,25 | 2,48 | 21,1 | 6,52 | 12,8 | 2,05 | 20 | 0,96 | 16,6 | 4,3 | 46 | 7,27 | 14,4 | 3,35 | 6,5 | 1,12 | 8,5 | 0,03 |  |  | 19,4 | 12,05 |
| RA major diameter,mm | 37 | 4,17 | 34 | 2,9 | 51 | 5,78 | 40,5 | 1,87 | 52 | 1,15 | 50,5 | 4,89 | 85,8 | 7,6 | 39 | 2,19 | 29 | 1,30 | 40,2 | 2,16 | 46 | 3,63 | 52,2 | 6,11 |
| Diastolic Dysf., Grade | II |  | II |  | III |  | III |  | III |  | II |  | III |  | II |  | III |  | III |  | III |  | II |  |
| E/A ratio | 1,7 |  | 1,8 |  | 2,6 |  | 2,8 |  | 4.2 |  | 1,6 |  | 3,2 |  | 1,8 |  | 2,5 |  | 2,5 |  | 2,8 |  | 1,8 |  |

EDD – end-diastolic diameter, EF – ejection fraction, ESD – end-systolic diameter, IVS – interventricular septum, LA – left atrium, LVPWd – left ventricular posterior wall thickness in diastole, RA – right atrium, RV – right ventricle.
